# Latent toxoplasmosis and olfactory functions of Rh positive and Rh negative subjects

**DOI:** 10.1371/journal.pone.0209773

**Published:** 2018-12-27

**Authors:** Jaroslav Flegr, Manfred Milinski, Šárka Kaňková, Martin Hůla, Jana Hlaváčová, Kateřina Sýkorová

**Affiliations:** 1 Division of Biology, Faculty of Science, Charles University in Prague, Prague, Czech Republic; 2 Applied Neurosciences and Brain Imagination, National Institute of Mental Health, Klecany, Czech Republic; 3 Department of Evolutionary Ecology, Max Planck Institute for Evolutionary Biology, Plön, Germany; Universita degli Studi di Parma, ITALY

## Abstract

**Backgrounds:**

The prevalence of toxoplasmosis is higher in schizophrenics than in the general population. It has been suggested that certain symptoms of schizophrenia, including changes in olfactory functions, are in fact symptoms of toxoplasmosis that can be easily detected in schizophrenics only due to the increased prevalence of toxoplasmosis in this population. Schizophrenics have impaired identification of odors and lower sensitivity of odor detection, however, no information about these parameters of non-schizophrenic *Toxoplasma*-infected subjects is available.

**Methods:**

Here we searched for differences in olfactory functions between 62 infected and 61 noninfected non-schizophrenic subjects using the case-controls experimental design.

**Results:**

The infected men scored better than the non-infected controls in the standard odor-identification test. The infected women rated all smells as more intensive while the infected men rated nearly all smells as less intensive. Infected women rated the pleasantness of the smell of the cat urine as higher than the non-infected women and the opposite was true for the men–in contrast, higher pleasantness of odor in infected men and lower in infected women were observed and described in the 2011 study. Toxoplasmosis, Rh, and toxoplasmosis-Rh interaction were not associated with the rated pleasantness of the smell of other stimuli. However, our sample contained only 17 Rh negative men and 30 Rh negative women. Therefore, all results concerning the main effects of Rh factor and the interaction with Rh factor must be considered only preliminary.

**Conclusions:**

Our results suggest that latent toxoplasmosis is associated with changes in the olfactory functions in humans; however, the observed changes differ from those observed in schizophrenics.

## Introduction

The protozoan parasite *Toxoplasma gondii* infects between 10–80% of the inhabitants of both developing and developed countries, depending on climate, hygienic and dietetic habits and the density of cats (the definitive host of *Toxoplasma*) in particular areas [1]. After a short phase of acute toxoplasmosis, the disease proceeds into the life-long latent phase. For a long time, this phase was considered more or less asymptomatic in immunocompetent individuals. However, within the last 15 years, more and more results suggest that the latent form of toxoplasmosis is associated with increased frequencies of various disorders, mainly but not exclusively with neuropsychiatric disorders, for review see [2,3]. It is widely accepted now that toxoplasmosis plays an important role in the etiology of schizophrenia. The prevalence of toxoplasmosis is higher in schizophrenia patients [4,5], especially in those with a continuous course of the disorder [6]. *Toxoplasma* codes two enzymes for the syntheses of dopamine [7], the neurotransmitter that plays a very important role in positive symptoms (hallucinations and delusions) of schizophrenia [8]. Latent toxoplasmosis is associated with the increased concentration of this neurotransmitter in the brain of infected rodent [9] and human [10] hosts. Increased level of dopamine could explain the higher intensity of positive symptoms and longer hospitalization in *Toxoplasma*-infected schizophrenics [11–13]. The specific morphological changes of the brain of schizophrenia patients, namely the decrease of grey matter density bilaterally located within the caudate, median cingulate, thalamus and occipital cortex and in the left cerebellar hemispheres can be detected only in those patients who are infected with *Toxoplasma* [14].

It has been suggested that some characteristics of schizophrenia patients could in fact be the results of the *Toxoplasma* infection, which is more frequently documented in schizophrenia patients than in the general population, rather than the direct effect of schizophrenia [15]. It is known, for example, that both schizophrenia patients and people with latent toxoplasmosis have an increased latency of startle reflex and a decreased effect of prepulse on the latency in the acoustic startle reflex inhibition test [15,16]. It has been also described that both toxoplasmosis and schizophrenia are associated with characteristic changes of olfactory functions [17,18]. *Toxoplasma*-infected subjects rate the pleasantness of smell of urine of the definitive host of *Toxoplasma*, the cat, but not the pleasantness of smell of urine of other four species, differently than the non-infected controls [19]. Namely, the *Toxoplasma* infected men rated this smell as more pleasant, which recalls the well-known “fatal attraction phenomenon” observed in infected rodents [20,21] and chimpanzee [22], while the *Toxoplasma*-infected women rate the smell of cat urine as less pleasant. The change of the natural fear of the smell of cat predators of animals towards an attraction to this smell after the *Toxoplasma* infection is considered to be the product of manipulation activity of the *Toxoplasma* aimed to increase the chance of its transmission from the intermediate to definitive host by predation. It has been shown that epigenetic modification (demethylation) of regulatory parts of certain genes in the medial amygdala is probably responsible for the observed behavioral changes [23]. The schizophrenia-associated effects seem to be less specific (however, the “fatal attraction” effect has never been studied in this population). A strong negative influence of schizophrenia on the performance of patients in the odor identification test has been reported in many studies [17,18]. A couple of studies also demonstrated schizophrenia-associated deficits in the detection threshold sensitivity, the odor discrimination and the olfactory recognition memory; for an excellent analytical review see [24]. These olfactory functions have not been studied in people with latent *Toxoplasma* infection.

In the present study, we searched for support for the hypothesis that the high prevalence of *Toxoplasma*-infected subjects (with olfactory function modified by the manipulation activity of the parasite) could be responsible for the reported olfactory deficits in schizophrenia patients. This hypothesis is based on the presumption that the subjects with latent *Toxoplasma* infection have impaired olfactory functions. This prediction, however, has never been experimentally tested. Here, we compared the performance of *Toxoplasma*-infected and *Toxoplasma*-free men and women in two variants of the odor identification test and investigated the differences in rating the intensity and pleasantness of four odor samples of animal origin (zibet, moschus, ambra, cat urine) and one of plant origin (jasmine). *Toxoplasma* has mostly opposite effects on behavior and personality of men and women and often different effects on Rh positive and Rh negative subjects [25]. The behavioral and performance effects of toxoplasmosis also mostly increase with time since the infection. Therefore, our *Toxoplasma*-infected and *Toxoplasma*-free populations were matched for sex, age, and Rh.

## Methods

### Subjects and procedure

Most of subjects were past students of biology who participated in various parasitological and evolutionary psychological studies that have run at the Faculty of Science in past 15 years. About one third were registered members of an internet community “Labbunnies” (Pokusni kralici in Czech, www.facebook.com/pokusnikralici). This community of people willing to take part in diverse evolutionary psychological experiments consists of subjects of various ages, education levels, occupations, and locations [26]. Within the last 4 years they were invited to come to the faculty or to other institutions in five large Czech cities for testing for toxoplasmosis and Rh phenotype. We invited by telephone the *Toxoplasma*-infected Rh negative and Rh positive subjects, as well as the same number of *Toxoplasma*-free controls balanced for sex, Rh, and age to come to the faculty to participate in a 45 minutes “sniffing experiment”. About 95% of subjects agreed to participate and about 75% were actually able to come to some of suggested appointments for testing. Men were tested within two weeks of January and two weeks of February 2017 and women within two weeks of March 2017. The participants were given the instructions not to use perfume, perfumed soap or perfumed deodorant on the day of the experiment and not to smoke or consume aromatic meals at least one hour before the session. The participants were not informed that the study was concerned with toxoplasmosis and this information was not written in the informed consent but was provided to the participants during a 5 minutes debriefing after the end of the experiment. After reading and signing the informed consent, the participants (alone and in their own tempo) rated the intensity and pleasantness of 10 odor samples in a quiet, ventilated room. To control for possible effect of the order of samples, one half of each subgroup rated the variant A (1 Jasmin, 2 Moschus, 3 Ambra, 4 Zibet, 5 neutral (only solvent), 6 Zibet, 7 Ambra, 8 Moschus, 9 Jasmin, 10 undiluted cat urine) and one half the variant B (1 Zibet, 2 Ambra, 3 Moschus, 4 Jasmin, 5 neutral (only solvent), 6 Jasmin, 7 Moschus, 8 Ambra, 9 Zibet, 10 undiluted cat urine). To rate the pleasantness of odors, the participants had to respond to the question “How pleasurable would it be to use perfume containing this ingredient and to smell like this?” using a paper form with a continuous graphic scale of the length 9 cm anchored with “very unpleasurable” on the left and “very pleasurable” on the right. To rate the intensity of odors, the participants had to respond to the question “How intensive is this smell?” using a second graphic scale of the length 9 cm anchored with “very intensive” on the left and “very weak/I do not smell anything” on the right. To study the unconscious smell preference by the chromassociation method [27], the raters were given 12 color pencils and were asked to choose which color was best suited to a particular smell. After finishing this part of the test, the participants were asked to complete an anamnestic questionnaire lasting ten minutes and containing 24 questions mostly on health, moods, and using perfumes and deodorants. They were also asked how many cigarettes they usually smoke per day (occasional smokers were recommended to use decimal numbers). In the final part of the session, an assistant of the same sex presented them, one after one, 12 sniffing stick of the Burghart odor identification test. In the free-recalling variant of the test the assistant asked the participant what he thinks this smell is, recorded the answer and then in the standard variant of the sniffing test presented him a card with four alternative answers to be chosen from. After finishing both variants of the odor identification test, the raters finished the second part of the chromassociation test, i.e., they arranged the pencils from the most favorite to the least favorite color. Then the assistant checked the completeness of the material and explained aims of the study to the participant. Each rater was invited separately for a particular time and got no reward for his participation, except a special badge issued for this occasion and sometimes the compensation of travel expenses for the non-Prague participants. Written informed consent was obtained from all subjects tested for toxoplasmosis (for “testing for various pathogens and use of the results of these tests in future scientific studies on effects of biological factors on physiology, personality and behavior”) and a second more specific written informed consent was obtained from all individual participants included in the study. The project, including the method of subjects’ recruitment, content and form of informed consents, and procedure of the data handling, has been carried out in accordance with the relevant guidelines and regulations and has been approved by the Institutional Review Board of the Faculty of Science, Charles University (Etická komise pro práci s lidmi a s lidským materiálem Přírodovědecké fakulty Univerzity Karlovy), approval number 2016/27.

### Material

The attractiveness of cat urine for *Toxoplasma*-infected and *Toxoplasma*-free subjects (and rodents and chimpanzees) is known to differ. In our recent historical past, nearly the whole human population was infected with *Toxoplasma*. It could therefore be expected that the attractiveness of some perfume ingredients of animal origin could also be the result of the *Toxoplasma* manipulation activities–of reprogramming of specific neural circuits in the amygdala. Therefore, we used three perfume ingredients of animal origin (zibet, moschus, ambra) and one of plant origin as a control (jasmine).

#### Perfume ingredients

The fragrances were natural products of the highest quality.

#### Zibet

1g Zibet (Zibet absolue Essencia 60-4250-0) in 80ml base mixing (70g Ethanol 99.8% + 10g Aqua dest.) pestled in Achat mortar, transferred to a Duran flask, 1 drop Tween 80 added, for 24h in shaking water quench (50°C/120rpm), then filtered (5–8μm). This preparation was diluted further 1:2.5: 1.6ml Zibet (1:65 as described above) + 2.4ml base mixing. The final dilution for the experiment was 1:162.5.

#### Ambra

1g Ambra (Ambra grau Essencia 60-2350-0) in 80ml base mixing, pestled in Achat mortar, transferred to a Duran flask, 1 drop Tween 80 (P4780 Sigma) added, for 24h in shaking water quench (50°C/120rpm), then filtered (5–8μm). This base preparation was not diluted further; the final dilution for the experiment was 1:65.

#### Moschus

0.8ml Original product (Moschuskörner 15% in Ethanol, Primavera 11128) + 3.2ml base mixing. The final dilution for the experiment was 1:5.

#### Jasmin

20μl Original product (Jasmin absolue, Primavera 10150) + 6.38ml base mixing. The final dilution for the experiment was 1:320.

Number-coded glass 3 ml vials each of which contained a 3 cm long smelling strip (as used in perfumology) that was fixed to the plug. Each strip was supplied with two drops (about 27 μl) of the respective perfume ingredient.

#### Cat urine

Aliquots of the mixture of urine from five female cats were frozen at -18°C. One week before the experiment, 10 μl of the mixture for women and 20 μl for men were pipetted on the strip of filtrating papers in the testing vial and the vials were again put into a -18°C freezer. A different volume of urine was used in men and women because the male and female sensitivity to the smell of cat urine probably differed. In fact, two of the six men did not identify the presence of any odor when 10 μl urine samples were used in our pilot experiment, while several women complained that the intensity and repulsiveness of smell of 10 μl samples was too high. At 10 minutes before the experiment, the vials were taken from the freezer and kept at room temperature until the beginning of the test.

### Odor identification tests

The Burghart Sniffin’ Sticks Screening 12 Test (Medisense, The Netherlands) with peppermint, fish, coffee, banana, orange, rose, lemon, pineapple, cinnamon, cloves, leather and liquorice odors was used in two variants of the odor identification test. Each participant was at first handed one sniffing stick by an assistant of the same sex (in the same sequence from 1 to 12) to try to identify a particular odor (the free-recalling variant of the test). Then, in the standard variant of the test, the assistant handed him/her a multiple choice form containing names of four different odors and the participant tried to identify the correct one. The assistant recorded the answers of the participants in both parts of the odor identification test and handed the participants the next sniffing stick.

### Immunological tests for *T*. *gondii* infection and Rh phenotype

All testing was performed at the National Reference Laboratory for Toxoplasmosis, National Institute of Public Health, Prague. The complement-fixation test (CFT), which determines the overall levels of IgM and IgG antibodies of particular specificity, and Enzyme-Linked Immunosorbent Assays (ELISA) (IgG ELISA: SEVAC, Prague) were used to detect the *T*. *gondii* infection status of the subjects. ELISA assay cut-point values were established using positive and negative standards according to the manufacturer’s instructions. In CFT, the titer of antibodies against *T*. *gondii* in sera was measured in dilutions between 1:8 and 1:1024. The subjects with CFT titers between 1:8 and 1:128 were considered *T*. *gondii* infected. Only subjects with clearly definitive results of CFT or IgG ELISA tests were diagnosed as *T*. *gondii*-infected or *T*. *gondii*-free. A standard agglutination method was used for Rh examination. A constant amount of anti-D serum (monoclonal human IgM blood grouping reagents; antiD clones MS-201, RUM-1, Millipore (UK) Ltd.) was added to a drop of blood on a white glass plate. Red cells of Rh-positive subjects were agglutinated within 2–5 minutes.

### Statistics

Before the analyses, the ratings on a graphical scale were measured and recorded independently by two persons and any inconsistencies were checked and corrected. They also calculated unconscious smell preference scores by computing the mean attractiveness score of color attributed by a particular rater to individual odors–the color the most attractive for the rater was coded as 12, the most unattractive as 1. To control for effects of sequence of presentation each perfume ingredient was present in two vials, therefore the arithmetic mean was calculated from these two values. Statistica v. 10.0 was used for all t-tests, Pearson’s and Spearman correlation test, ANCOVA, repeated measure ANCOVA, and for testing of the tests’ assumptions. Partial Kendall correlation with age as the confounding variable was computed using an Excel sheet available at http://web.natur.cuni.cz/flegr/programy.php. Some variables, e.g. the frequencies of smoking, had highly asymmetric distribution, therefore we used both parametric and nonparametric tests for their analysis; however, the results were qualitatively the same. The Bonferroni’s correction was used for the correction for multiple tests. The data file is available at 10.6084/m9.figshare.6291512.

### Terminological notes

For the sake of clarity, we abbreviated “*Toxoplasma* seropositivity” to “toxoplasmosis”in the Discussion and to “toxo” in the description of our statistical models. Also, the statistical relations between (formally) dependent and (formally) independent variables are called “effects,” despite the fact that the real causal relation between these variables can be different or even non-existent.

## Results

### a) Descriptive statistics

The final sample consisted of 60 women (age: 28.0, SD 4.46) and 63 men (age: 28.1, SD 5.69). In the women, 30 subjects were *Toxoplasma*-infected (15 Rh-negative and 15 Rh-positive) and 30 *Toxoplasma*-free (15 Rh-negative and 15 Rh-positive). In the 63 men, 32 subjects were *Toxoplasma*-infected (9 Rh-negative and 23 Rh-positive) and 31 were *Toxoplasma*-free (8 Rh-negative and 23 Rh-positive). No significant differences in age existed between the men and women, *Toxoplasma*-infected and *Toxoplasma*-free, Rh-negative or Rh-positive groups of subjects (all t-test p values > 0.20).

### b) Effect of toxoplasmosis on the identification of odors

**Fig 1:**
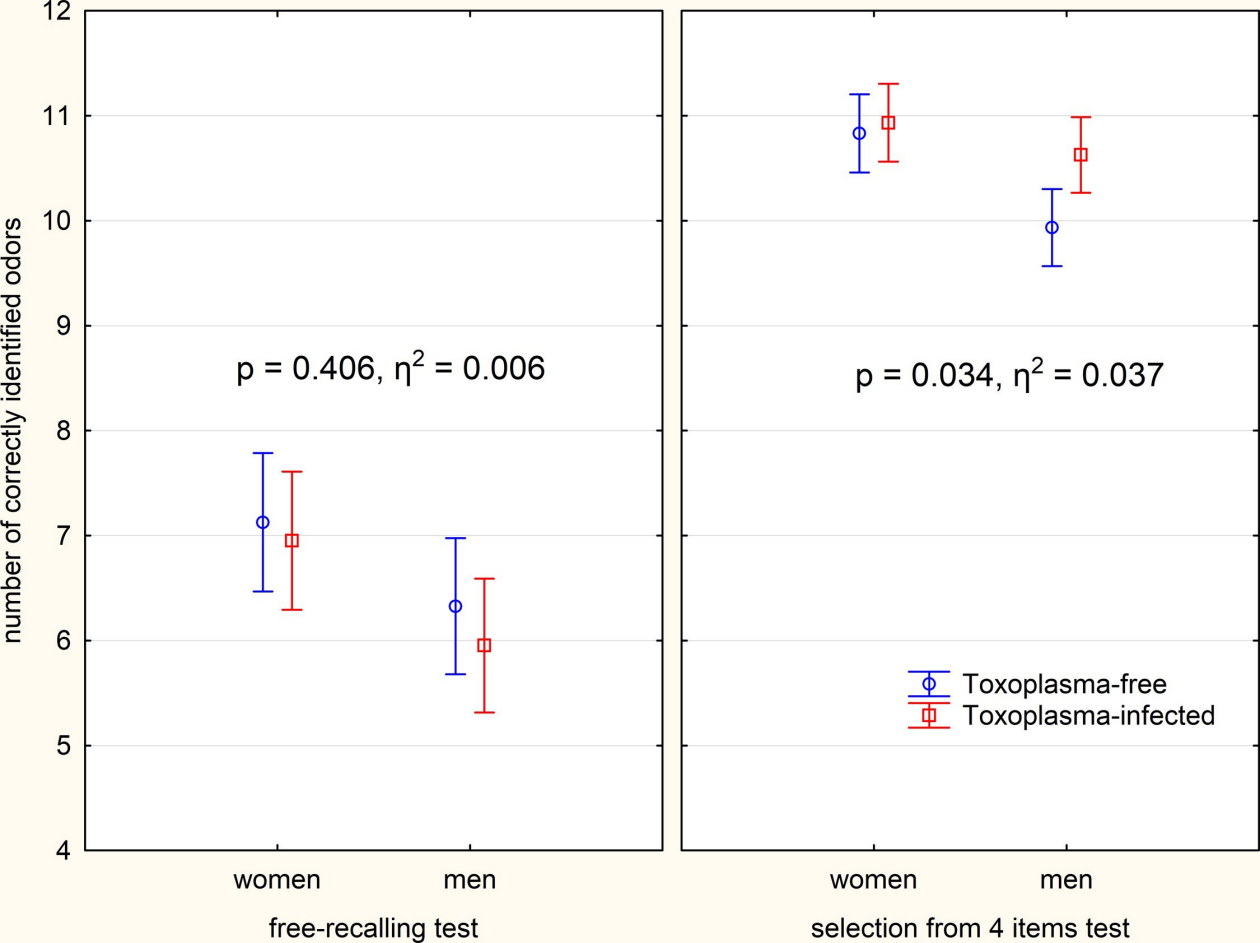
Differences of abilities to recognize odors in the *Toxoplasma*-free and *Toxoplasma*-infected subjects. The error bars show 95% confidence intervals, the numbers the results of ANCOVA test–the association between toxoplasmosis and performance of subjects in particular tests.

Women recognized more odors than men in both variants of the test (ANCOVA, independent variables sex (binary), age (continuous): p = 0.01, η^2^ = 0.090). The *Toxoplasma*-infected subjects identified more odors in the standard variant of the test (selection of the answer from four different options) and fewer odors in the first free-recalling variant of the test (Fig 1).

The effect of the interaction toxoplasmosis-type of the test was measured by repeated measures ANCOVA with the number of correctly identified odors in the free-recalling and standard odor identification tests as repeated dependent variables and the *Toxoplasma* infection (Toxo, binary), Rh phenotype (Rh, binary) and age as independent variables. This effect was significant when the variable Rh was present (p = 0.030, η^2^ = 0.041) or absent (p = 0.016, η^2^ = 0.048) in the model. A separate ANCOVA analysis of the two variants of the test showed that the effect of toxoplasmosis was significant in the standard variant of the test (Rh included: p = 0.048, η^2^ = 0.034); Rh not included: p = 0.034, η^2^ = 0.037) and not significant in the free-recalling variant of the test (Rh included: p = 0.473, η^2^ = 0.005; Rh not included: p = 0.406, η^2^ = 0.006). Partial Kendall correlation with the age as a confounding variable showed that the positive effect of toxoplasmosis in the standard variant of the test was significant for all participants (partial Tau = 0.140, p = 0.021) and men (partial Tau = 0.242, p = 0.005), but non significant for women (partial Tau = 0.078, p = 0.379).

### c) Effect of toxoplasmosis on rated intensity of odor

A negative correlation existed between the rated intensity and pleasantness of particular smells as well as between the age or frequency of smoking and the rated intensity of smell. Also, positive correlation existed between frequency of smoking and rated pleasantness of smell, see Table 1. Therefore, we included the covariates of the age of subjects and the intensity of smoking (continuous) into all analyses, and the age of subjects, intensity of smoking and intensity of smell rated by the same subject into all ANCOVA analyses in which the effects of toxoplasmosis on pleasantness of smell was studied.

**Table 1:** Correlation between rated pleasantness and intensity of particular odors.

	age	Cat intensity	Jasmin intensity	Moschus intensity	Ambra intensity	Zibet intensity	neutral intensity	smoking frequency
age	1.00	-0.01	-0.13	-0.10	-0.11	**-0.23**	**-0.31**	-0.11
Cat	0.02	**-0.67**	**-0.21**	**-0.18**	-0.04	-0.05	-0.15	0.11
Jasmin	**-0.19**	**-0.21**	**-0.28**	-0.10	-0.13	-0.05	**-0.21**	0.15
Moschus	0.00	-0.06	-0.12	**-0.41**	-0.07	-0.12	-0.15	0.03
Ambra	-0.05	-0.05	-0.16	-0.10	**-0.53**	**-0.42**	-0.12	**0.19**
Zibet	0.04	0.00	**-0.22**	**-0.24**	**-0.47**	**-0.65**	-0.16	0.11
neutral	-0.02	-0.10	-0.08	-0.07	-0.17	**-0.18**	**-0.29**	0.15
smoking	-0.11	-0.06	0.07	0.08	**-0.24**	-0.10	-0.02	1.00

Table shows Spearman R for the correlations of intensities (columns) and pleasantness (rows) of particular odors (and age and frequency of smoking). Significant coefficients (p<0.05) are printed in bold.

**Fig 2:**
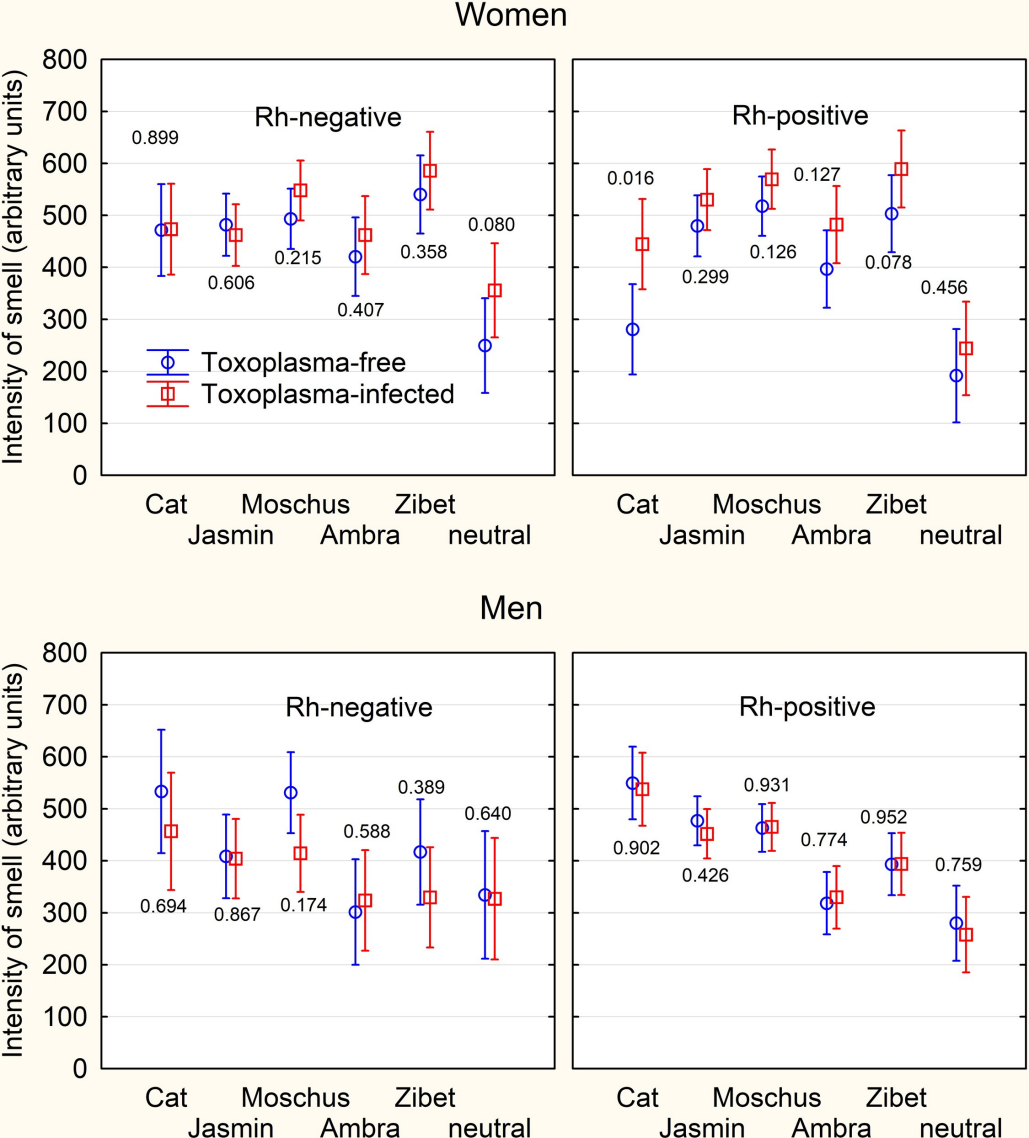
Difference in the intensities of smells attributed by women and men to particular odors. The error bars show 95% confidence intervals, the numbers the results of ANCOVA test–the p-value of the association of toxoplasmosis with the reported intensity of smell of particular samples. No result remained significant after the correction for multiple tests.

Repeated measure ANCOVA with attributed intensity of 6 odors (including the neutral sample–the empty vial) as repeated measures and toxoplasmosis, sex, Rh, age, and intensity of smoking as the independent variables revealed the significant effect of toxoplasmosis-sex interaction (p = 0.015, η^2^ = 0.051), age (p = 0.005, η^2^ = 0.069), sex (p = 0.012, η^2^ = 0.055), age-type of odor interaction (p = 0.001, η^2^ = 0.035), sex-type of odor interaction (p < 0.0005, η^2^ = 0.135), smoking-type of odor interaction (p = 0.006, η^2^ = 0.028), and Rh-type of odor interaction (p = 0.017, η^2^ = 0.024). The *post-hoc* tests showed that the *Toxoplasma*-infected women rated the intensity of the smell of all odors as significantly or non-significantly higher than the *Toxoplasma*-infected women (cat: partial Tau = 0.176, p = 0.047; jasmin: partial Tau = 0.060, p = 0.501; moschus: partial Tau = 0.179, p = 0.043; ambra: partial Tau = 0.164, p = 0.063; zibet: partial Tau = 0.191, p = 0.031; neutral: partial Tau = 0.180, p = 0.042), while *Toxoplasma*-infected men rated all except one of the intensities as non-significantly lower than the *Toxoplasma*-infected men (cat: partial Tau = -0.066, p = 0.444; jasmin: partial Tau = -0.081, p = 0.350; moschus: partial Tau = -0.096, p = 0.269; ambra: partial Tau = 0.052, p = 0.547; zibet: partial Tau = -0.086, p = 0.318; neutral: partial Tau = -0.053, p = 0.539), see the Fig 2.

### d) Effects of toxoplasmosis on rated pleasantness of odors

**Fig 3:**
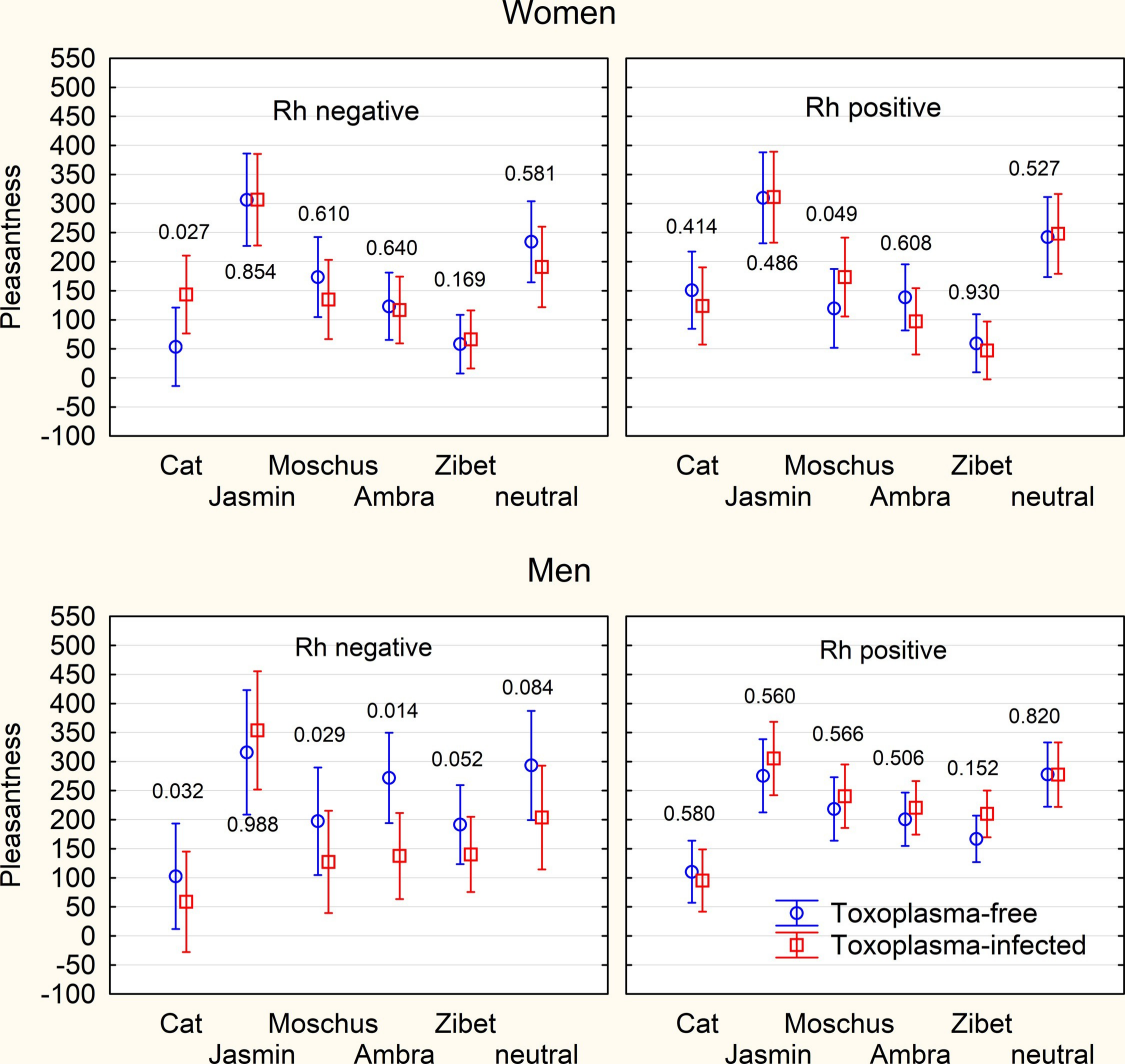
Difference in pleasantness attributed to particular odors by *Toxoplasma*-free and *Toxoplasma*-infected raters. The figure shows mean pleasantness of odors in arbitrary units measured as the responses of the raters to the question “How pleasurable would it be to use perfume containing this ingredient and to smell like this?” (graphic scale, 9 cm). The spreads shows 95% confidence interval, the numbers the results of ANCOVA test–the p-value of the association of toxoplasmosis with the reported pleasantness of smell of particular samples. No result remained significant after the correction for multiple tests.

Repeated measure ANCOVA with attributed pleasantness of six odors (including the neutral sample) as repeated measures and toxoplasmosis, sex, Rh, intensity of smoking and age as an independent variables revealed only significant effects of sex (p = 0.009, η^2^ = 0.059), type of odor (p < 0.0005, η^2^ = 0.056), sex-type of odor interaction (p < 0.0005, η^2^ = 0.060), and a nearly significant effect of smoking (p = 0.056, η^2^ = 0.032), Fig 3. No other effect was significant and the same was true for the simpler model without Rh. The results of separate analyses for particular odors with toxoplasmosis, sex, age and the intensity of particular smell as the independent variables provided a significant effect of toxoplasmosis-sex interaction on the smell of cat urine (p = 0.020, η^2^ = 0.046). The models that also included Rh and its interactions with other independent binary variables showed significant effects of toxoplasmosis-sex interaction for the cat urine (p = 0.015, η^2^ = 0.051), toxoplasmosis-Rh interaction for the moschus (p = 0.015, η^2^ = 0.051), and toxoplasmosis-sex-Rh interaction for the ambra (p = 0.030, η^2^ = 0.041) and the zibet (p = 0.036, η^2^ = 0.039). After the correction for multiple tests, however, no main effect of toxoplasmosis or of interaction with toxoplasmosis was significant in any model.

**Fig 4:**
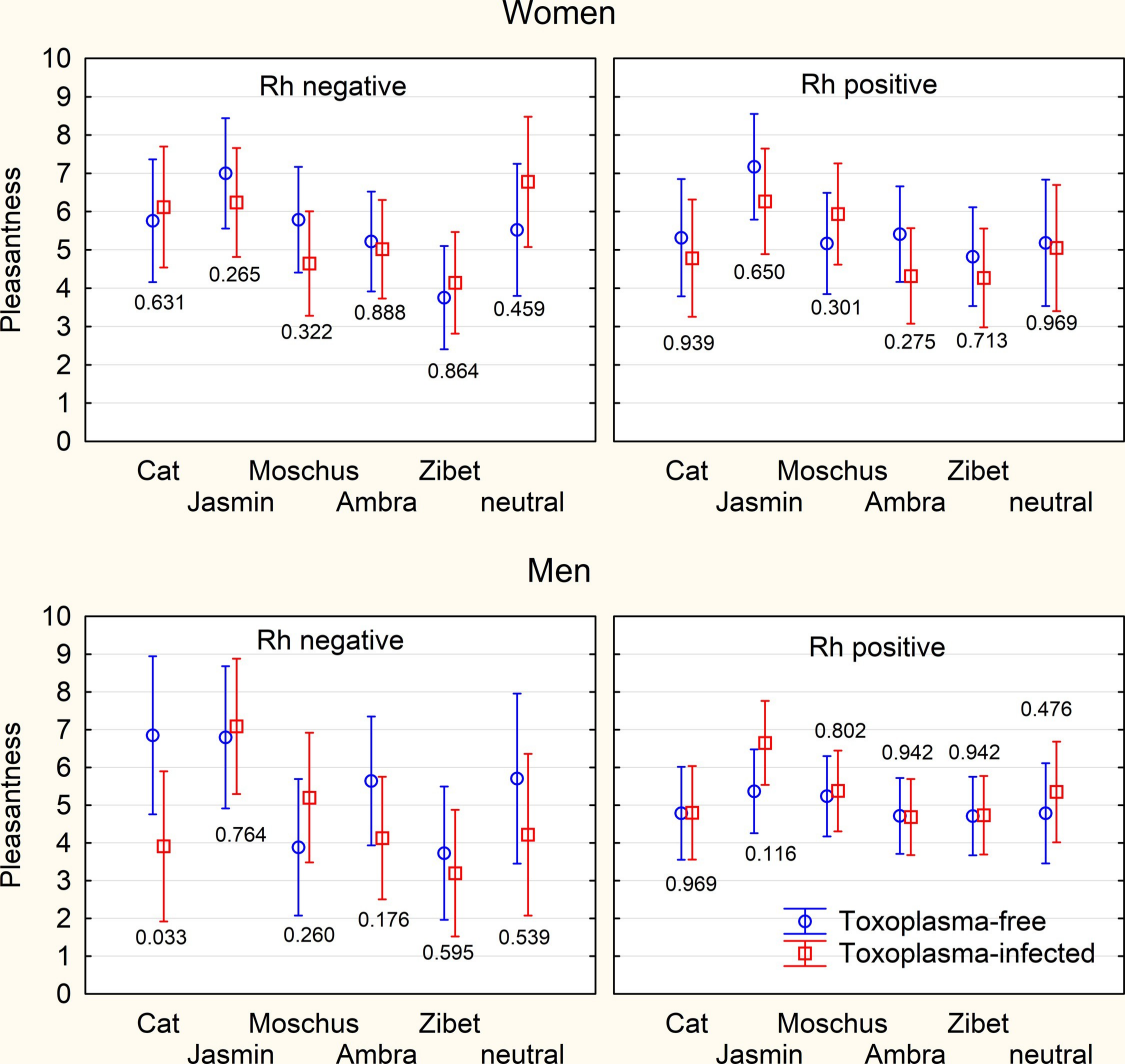
Pleasantness of odors estimated by the chromassociation method. The figure shows mean pleasantness of color (scale 1–12) attributed by the individual raters to particular odor. The spreads shows 95% confidence interval, the numbers the results of ANCOVA test–the p-value of the association of toxoplasmosis with the pleasantness of smell of particular samples measured with the chromassociation method. No result remained significant after the correction for multiple tests.

Identical analyses have been repeated with the pleasantness of odors estimated with chromatic association method. Except the effect of the type of odor (p = 0.001, η^2^ = 0.027), no other effects were significant, Fig 4.

## Discussion

We found significantly better performance of *Toxoplasma*-infected subjects, especially the men, in the standard odor identification test. *Toxoplasma*-infected women rated all smells as more intensive while the *Toxoplasma*-infected men rated nearly all smells as less intensive. Infected female raters rated the pleasantness of the smell of the cat urine as higher than the non-infected female raters and the opposite was true for the male raters. We found no evidence for the effect of toxoplasmosis on the rated pleasantness of the smell of jasmin, moschus, ambra, and zibet.

The better performance of the *Toxoplasma*-infected subjects in the odor identification test and the higher scores of intensity of smell attributed to the odors in women was unexpected. It is, however, possible that the infected subjects have in fact unchanged olfactory functions but try more hardly to identify the odor during the test and express higher willingness to rate the intensity of smell as higher. For example, infected women are known to express higher affectothymia, warmth, and cooperativeness than non-infected women [28,29]. All these traits could results in larger effort invested into the test and in higher ratings attributed to particular odors. Infected men express higher concentration of testosterone [30] and therefore higher competitiveness, which could also result in higher number of correctly identified odors, and higher suspiciousness [31], which could result in their lower ratings of the intensity of odors–they could suspect that some vials with low-intensity smell samples are empty).

The opposite effect of toxoplasmosis on the rated pleasantness of cat urine in men and women has been already described. However, in the previous study the infection increased the rated pleasantness of the smell in men and decreased it in women [19]. The effect size of the interaction was similar in the original (μ^2^ = 0.057) [19] and the current study (μ^2^ = 0.046). It is known that the relationship between the attractiveness of the smell of cat urine for the *Toxoplasma*-infected rodents has a reverse U shape [32]. It is high for a medium concentration of urine but low for very low and for very high concentrations of urine. Moreover, recently published results showed that the direction and size of the effect of toxoplasmosis on pleasantness of the cat urine smell could depend on some unknown environmental factor(s) [33]. It will be necessary to include several concentrations of cat urine into future studies and examine in detail the effects of sex-toxoplasmosis-concentration interaction. Also, the published data concerning the fatal attraction phenomenon should be approached very carefully. It is probable that the authors are more willing to publish (and could do so more easily) the results that are in an agreement with the already published results and with the theory (the attraction to cat smell) than the results showing an opposite-direction shift in behavioral response of infected hosts (the cat smell avoidance). The adjustment of concentration of cat urine or even selective reporting of the results only for females or only for males can result in a clear but possibly untrue picture of the complex phenomenon of fatal attraction that current scientific literature shows.

Formally, we did not detect any effect of toxoplasmosis on the rating of the pleasantness of odors of four perfume ingredients as all effects disappeared after the correction for multiple tests. There were just minor differences between the patterns when the pleasantness of odors was rated by the subjects or measured with the chromassociation test, except relative higher scores for cat urine obtained with the chromassociation tests. Based on results obtained with our set of wide spread natural ingredients we conclude that toxoplasmosis has no significant effect on the preference for these perfume ingredients to be used for themselves. The preference for these ingredients has been shown to correlate with the possession of specific MHC immune genes [34] and MHC genes play a role in resistance of intermediate hosts to toxoplasmosis [35,36]. The absence of significant effects of toxoplasmosis on the pleasantness of odors suggests that different MHC genes play a role in the preference of perfumes and in the resistance to *Toxoplasma*.

Absence of any Rh-toxoplasmosis interaction, can be, at least partly, caused by a relatively low number of participants of the Rh-related part of the study, specifically the low number of Rh-negative men (8 *Toxoplasma*-free and 9 *Toxoplasma*-infected). Within past 25 years, we have tested many thousands of volunteers for toxoplasmosis and about three thousands of them are willing to continue to participate in our experiments. However, the number of men is lower than number of women due to unbalanced sex ratio in local biology students. Further, the prevalence of toxoplasmosis in current students decreased to less than 10% and the frequency of Rh-negative persons in the Czech population is about 16%. Therefore, we were not able to convince more *Toxoplasma*-infected, Rh negative men to come to our lab for testing.

Rh factor has a very strong effect on the response of human organisms to *Toxoplasma* infection. Some effects of toxoplasmosis are much stronger in Rh negative subjects and some can be observed only in this population or in Rh-positive homozygotes [37–40]. In the present study, we did not detect any interaction of Rh-toxoplasmosis that would survive the correction for multiple tests. However, we found that the Rh negative and Rh positive subjects differ in the intensity and possibly also pleasantness of odor attributed to different perfume ingredients, see Fig 2. This anecdotal observation could have some practical implication for the perfume industry and would deserve future attention.

The main topic of the present study was the effect of toxoplasmosis on the olfactory function of healthy men and women. However, we found some unrelated known or unknown phenomena that deserve future attention. Our data suggest that smoking has positive influence on the on rating pleasantness of all odors and negative influence on rating intensity of some, but not all odors. The subjectively rated intensity to, as well as the pleasantness of, some odors differs between men and women. The women rated the pleasantness of the smell of moschus, ambra and especially zibet very low not only in comparison with the smell of a neutral sample but even in comparison with the smell of the cat urine. During the rating sessions, the participants were requested to answer the question “How pleasurable would it be to use a perfume containing this ingredient and to smell like this?” While men strongly disliked the prospect of smelling like cat urine, the women rated the smell of cat urine as more pleasant than that of zibet, the smell of which is subjectively reminiscent of cat urine. This seems to be in conflict with the observation showing that zibet is contained in 31%, i.e. 152 of 489, of perfumes for women but only in 6%, i.e. 23 of 377, of perfumes for men (data analyzed from H&R Fragrance Guide—Fragrances on the international Market. Glöss, Hamburg 1995). The most obvious explanation, namely that the perfumes for men are mainly chosen by women and vice versa, is almost certainly false–perfumologists regard it as a fact that both women and men choose perfumes for themselves and usually do not use any other perfumes chosen by someone else, see e.g. [41], Perfumery–The Psychology and Biology of fragrance. Steve Van Toller, George H. Dodd (eds). Chapman & Hall, London, 1994).

### Limitations

The most important limitation is a rather low number of Rh negative men participating in the study. Due to this limitation, all positive and negative results concerning the effects of and the interaction with Rh factor must be considered only preliminary. We used only the small variant of the sniffing test. Many women identified all 12 samples in the standard variant of the test. Therefore, the sensitivity of the method was decreased due to ceiling effect in women and also partly in men. We used only one concentration (and one sample of) the cat urine in the set of samples. In future studies, both undiluted and diluted samples of cat urine as well as a sample of urine of an animal that is not the definitive hosts of *Toxoplasma gondii* should be used.

## Conclusions

Our data confirmed that latent toxoplasmosis has some effects on the olfactory function of humans. The strength of the effects is mostly low (η^2^ < 0.06), which corresponded to strength of other effects of toxoplasmosis on human performance and behavior. In contrast to other toxoplasmosis-associated effects, no significant differences were observed between Rh negative and Rh positive infected subjects. In contrast, an opposite effect of toxoplasmosis on the olfactory functions was observed in men and women, the phenomenon which has been described earlier for many behavioral and personality traits [28,31]. This suggests that the observed differences, possibly including the observed analogy of the fatal attraction phenomenon [19], are not the primary effects of toxoplasmosis on the olfactory system but rather the secondary effects of toxoplasmosis-associated personality changes. According to the stress coping hypothesis [29], the opposite effects of toxoplasmosis on the behavior and personality of men and women can be explained by the opposite behavioral reaction of men and women to the chronic stress that is caused by the life-long parasitic infection. It is known that stressed men use more individualistic and antisocial (e.g. aggressive, hostile) forms of coping with stress, while stressed women are more likely to seek and provide social support. The observed difference in performance of infected and non-infected men and women could be just secondary effects of such coping.

We detected some differences in the olfactory functions of infected and non-infected subjects, however, the observed changes seems to differ from the changes described in schizophrenia patients. While schizophrenia patients have an impaired ability to identify odors, the *Toxoplasma* infected subjects performed better in the standard odor identification test. Therefore, our results are in conflict with our main hypothesis suggesting that the observed changes in olfactory performance of schizophrenia patients could be an experimental artifact caused by higher prevalence of *Toxoplasma*-infected subjects in the schizophrenia patients than in the non-clinical population [15].

## References

[1] Tenter AM, Heckeroth AR, Weiss LM (2000) *Toxoplasma gondii*: from animals to humans. International Journal for Parasitology 30: 1217–1258. 11113252 10.1016/s0020-7519(00)00124-7 PMC3109627

[2] Flegr J, Prandota J, Sovickova M, Israili ZH (2014) Toxoplasmosis—A global threat. Correlation of latent toxoplasmosis with specific disease burden in a set of 88 countries. PLoS ONE 9. 10.1371/journal.pone.0090203 PMC3963851 24662942

[3] Sutterland AL, Fond G, Kuin A, Koeter MW, Lutter R, van Gool T, et al (2015) Beyond the association. *Toxoplasma gondii* in schizophrenia, bipolar disorder, and addiction: systematic review and meta-analysis. Acta Psychiatrica Scandinavica 132: 161–179. 10.1111/acps.12423 25877655

[4] Torrey EF, Bartko JJ, Lun ZR, Yolken RH (2007) Antibodies to *Toxoplasma gondii* in patients with schizophrenia: A meta-analysis. Schizophrenia Bulletin 33: 729–736. 10.1093/schbul/sbl050 17085743 PMC2526143

[5] Torrey EF, Bartko JJ, Yolken RH (2012) *Toxoplasma gondii* and other risk factors for schizophrenia: An update. Schizophrenia Bulletin 38: 642–647. 10.1093/schbul/sbs043 22446566 PMC3329973

[6] Celik T, Kartalci S, Aytas O, Akarsu GA, Gozukara H, Unal S (2015) Association between latent toxoplasmosis and clinical course of schizophrenia—continuous course of the disease is characteristic for *Toxoplasma gondii*-infected patients. Folia Parasitologica 62. 10.14411/fp.2015.015 25960559

[7] Gaskell EA, Smith JE, Pinney JW, Westhead DR, McConkey GA (2009) A unique dual activity amino acid hydroxylase in *Toxoplasma gondii* PloS ONE 4: e4801 10.1371/journal.pone.0004801 19277211 PMC2653193

[8] Willner P (1997) The dopamine hypothesis of schizophrenia: current status, future prospects. International Clinical Psychopharmacology 12: 297–308. 9547131 10.1097/00004850-199711000-00002

[9] Prandovszky E, Gaskell E, Martin H, Dubey JP, Webster JP, McConkey GA (2011) The neurotropic parasite *Toxoplasma gondii* increases dopamine metabolism. PLoS ONE 6: e23866 10.1371/journal.pone.0023866 21957440 PMC3177840

[10] Flegr J, Preiss M, Klose J, Havlíček J, Vitáková M, Kodym P (2003) Decreased level of psychobiological factor novelty seeking and lower intelligence in men latently infected with the protozoan parasite *Toxoplasma gondii*. Dopamine, a missing link between schizophrenia and toxoplasmosis? Biological Psychology 63: 253–268. 12853170 10.1016/s0301-0511(03)00075-9

[11] Wang HL, Wang GH, Li QY, Shu C, Jiang MS, Guo Y (2006) Prevalence of *Toxoplasma* infection in first-episode schizophrenia and comparison between *Toxoplasma*-seropositive and *Toxoplasma*-seronegative schizophrenia. Acta Psychiatrica Scandinavica 114: 40–48. 10.1111/j.1600-0447.2006.00780.x 16774660

[12] Yolken RH, Dickerson FB, Torrey EF (2009) *Toxoplasma* and schizophrenia. Parasite Immunology 31: 706–715. 10.1111/j.1365-3024.2009.01131.x 19825110

[13] Holub D, Flegr J, Dragomirecka E, Rodriguez M, Preiss M, Novak T, et al (2013) Differences in onset of disease and severity of psychopathology between toxoplasmosis-related and toxoplasmosis-unrelated schizophrenia. Acta Psychiatrica Scandinavica 127: 227–238. 10.1111/acps.12031 23126494

[14] Horacek J, Flegr J, Tintera J, Verebova K, Spaniel F, Novak T, et al (2012) Latent toxoplasmosis reduces gray matter density in schizophrenia but not in controls: Voxel-based-morphometry (VBM) study. World Journal of Biological Psychiatry 13: 501–509. 10.3109/15622975.2011.573809 21599563

[15] Priplatova L, Sebankova B, Flegr J (2014) Contrasting effect of prepulse signals on performance of *Toxoplasma*-infected and *Toxoplasma*-free subjects in an acoustic reaction times test. PLoS ONE 9. 10.1371/journal.pone.0112771 PMC4226587 25384036

[16] Pearce BD, Hubbard S, Rivera HN, Wilkins PP, Fisch MC, Hopkins MH, et al (2013) *Toxoplasma gondii* exposure affects neural processing speed as measured by acoustic startle latency in schizophrenia and controls. Schizophrenia Research 150: 258–261. 10.1016/j.schres.2013.07.028 23953218 PMC3786776

[17] Rupp CI (2010) Olfactory function and schizophrenia: an update. Current Opinion in Psychiatry 23: 97–102. 10.1097/YCO.0b013e328336643f 20051859

[18] Kamath V, Turetsky BI, Moberg PJ (2011) Identification of pleasant, neutral, and unpleasant odors in schizophrenia. Psychiatry Research 187: 30–35. 10.1016/j.psychres.2010.12.011 21239063 PMC3073768

[19] Flegr J, Lenochová P, Hodný Z, Vondrová M (2011) Fatal attraction phenomenon in humans: cat odour attractiveness increased for *Toxoplasma*-infected men while decreased for infected women. PLoS Neglected Tropical Diseases 5: e1389 10.1371/journal.pntd.0001389 22087345 PMC3210761

[20] Berdoy M, Webster JP, Macdonald DW (2000) Fatal attraction in rats infected with *Toxoplasma gondii*. Proceedings of the Royal Society B-Biological Sciences 267: 1591–1594. 10.1098/rspb.2000.1182 PMC1690701 11007336

[21] Vyas A, Kim SK, Giacomini N, Boothroyd JC, Sapolsky RM (2007) Behavioral changes induced by *Toxoplasma* infection of rodents are highly specific to aversion of cat odors. Proceedings of the National Academy of Sciences of the United States of America 104: 6442–6447. 10.1073/pnas.0608310104 17404235 PMC1851063

[22] Poirotte C, Kappeler PM, Ngoubangoye B, Bourgeois S, Moussodji M, Charpentier MJE (2016) Morbid attraction to leopard urine in *Toxoplasma*-infected chimpanzees. Current Biology 26: R98–R99. 10.1016/j.cub.2015.12.020 26859275

[23] Dass SAH, Vyas A (2014) *Toxoplasma gondii* infection reduces predator aversion in rats through epigenetic modulation in the host medial amygdala. Molecular Ecology 23: 6114–6122. 10.1111/mec.12888 25142402

[24] Moberg PJ, Agrin R, Gur RE, Gur RC, Turetsky BI, Doty RL (1999) Olfactory dysfunction in schizophrenia: A qualitative and quantitative review. Neuropsychopharmacology 21: 325–340. 10.1016/S0893-133X(99)00019-6 10457530

[25] Flegr J (2013) Influence of latent *Toxoplasma* infection on human personality, physiology and morphology: pros and cons of the *Toxoplasma*-human model in studying the manipulation hypothesis. Journal of Experimental Biology 216: 127–133. 10.1242/jeb.073635 23225875

[26] Flegr J, Horacek J (2017) *Toxoplasma*-infected subjects report an obsessive-compulsive disorder diagnosis more often and score higher in obsessive-compulsive inventory. European Psychiatry 40: 82–87. 10.1016/j.eurpsy.2016.09.001 27992837

[27] Ščepichin V, Ščepichinová J, Malá K (1995) Chromatický asociační experiment. Trnávka: Jindřich Horkel Elektronik Test.

[28] Flegr J, Zitkova S, Kodym P, Frynta D (1996) Induction of changes in human behaviour by the parasitic protozoan *Toxoplasma gondii*. Parasitology 113: 49–54. 8710414 10.1017/s0031182000066269

[29] Lindová J, Kuběna AA, Šturcová A, Křivohlavá R, Novotná M, Rubešová A, et al (2010) Pattern of money allocation in experimental games supports the stress hypothesis of gender differences in *Toxoplasma gondii*-induced behavioural changes. Folia Parasitologica 57: 136–142. 20608476

[30] Flegr J, Lindová J, Kodym P (2008) Sex-dependent toxoplasmosis-associated differences in testosterone concentration in humans. Parasitology 135: 427–431. 10.1017/S0031182007004064 18205984

[31] Lindová J, Novotná M, Havlíček J, Jozífková E, Skallová A, Kolbeková P, et al (2006) Gender differences in behavioural changes induced by latent toxoplasmosis. International Journal for Parasitology 36: 1485–1492. 10.1016/j.ijpara.2006.07.008 16978630

[32] Vyas A, Kim SK, Sapolsky RM (2007) The effects of *Toxoplasma* infection on rodent behavior are dependent on dose of the stimulus. Neuroscience 148: 342–348. 10.1016/j.neuroscience.2007.06.021 17683872 PMC2430144

[33] Abdulai-Saiku S, Hegde A., Vyas A., Mitra R. (2018) Effects of stress or infection on rat behavior show robust reversals due to environmental disturbance. F1000 Research 6:2097: 1–14. 10.12688/f1000research.13171.1 PMC5782406 29416851

[34] Milinski M, Wedekind C (2001) Evidence for MHC-correlated perfume preferences in humans. Behavioral Ecology 12: 140–149.

[35] Mack DG, Johnson JJ, Roberts F, Roberts CW, Estes RG, David C, et al (1999) HLA-class II genes modify outcome of *Toxoplasma gondii* infection. International Journal for Parasitology 29: 1351–1358. 10579423 10.1016/s0020-7519(99)00152-6

[36] Fux B, Rodrigues CV, Portela RW, Silva NM, Su CL, Sibley D, et al (2003) Role of cytokines and major histocompatibility complex restriction in mouse resistance to infection with a natural recombinant strain (type I-III) of *Toxoplasma gondii*. Infection and Immunity 71: 6392–6401. 10.1128/IAI.71.11.6392-6401.2003 14573660 PMC219541

[37] Novotná M, Havlíček J, Smith AP, Kolbeková P, Skallová A, Klose J, et al (2008) *Toxoplasma* and reaction time: Role of toxoplasmosis in the origin, preservation and geographical distribution of Rh blood group polymorphism. Parasitology 135: 1253–1261. 10.1017/S003118200800485X 18752708

[38] Flegr J, Klose J, Novotná M, Berenreitterová M, Havlíček J (2009) Increased incidence of traffic accidents in *Toxoplasma*-infected military drivers and protective effect RhD molecule revealed by a large-scale prospective cohort study. BMC Infectious Diseases 9: art. 72. 10.1186/1471-2334-9-72 PMC2692860 19470165

[39] Flegr J, Novotná M, Lindová J, Havlíček J (2008) Neurophysiological effect of the Rh factor. Protective role of the RhD molecule against *Toxoplasma*-induced impairment of reaction times in women. Neuroendocrinology Letters 29: 475–481. 18766148

[40] Kaňková Š, Šulc J, Flegr J (2010) Increased pregnancy weight gain in women with latent toxoplasmosis and RhD-positivity protection against this effect. Parasitology 137: 1773–1779. 10.1017/S0031182010000661 20602855

[41] (1994) Perfumery–The psychology and biology of fragrance; Van Toller S, Dodd GH, editors. London: Chapman & Hall.

